# Relationships of the early insulin secretory response and oral disposition index with gastric emptying in subjects with normal glucose tolerance

**DOI:** 10.14814/phy2.13122

**Published:** 2017-02-27

**Authors:** Chinmay S. Marathe, Christopher K. Rayner, Kylie Lange, Michelle Bound, Judith Wishart, Karen L. Jones, Steven E. Kahn, Michael Horowitz

**Affiliations:** ^1^ Discipline of MedicineUniversity of AdelaideRoyal Adelaide HospitalAdelaideAustralia; ^2^ Centre of Research Excellence (CRE) in Translating Nutritional Science to Good HealthUniversity of AdelaideAdelaideAustralia; ^3^ Division of MetabolismEndocrinology and NutritionVA Puget Sound Health Care System and University of WashingtonSeattleWashington

**Keywords:** Gastric emptying, insulin secretory response, oral disposition index, oral glucose tolerance test

## Abstract

The oral disposition index, the product of the early insulin secretory response during an oral glucose tolerance test and insulin sensitivity, is used widely for both the prediction of, and evaluation of the response to interventions, in type 2 diabetes. Gastric emptying, which determines small intestinal exposure of nutrients, modulates postprandial glycemia. The aim of this study was to determine whether the insulin secretory response and the disposition index (DI) related to gastric emptying in subjects with normal glucose tolerance. Thirty‐nine subjects consumed a 350 mL drink containing 75 g glucose labeled with ^99m^Tc‐sulfur colloid. Gastric emptying (by scintigraphy), blood glucose (G) and plasma insulin (I) were measured between *t* = 0–120 min. The rate of gastric emptying was derived from the time taken for 50% emptying (*T*_50_) and expressed as kcal/min. The early insulin secretory response was estimated by the ratio of the change in insulin (∆I_0–30_) to that of glucose at 30 min (∆G_0–30_) represented as ∆I_0–30_/∆G_0–30_. Insulin sensitivity was estimated as 1/fasting insulin and the DI was then calculated as ∆I_0–30_/∆G_0–30_ × 1/fasting insulin. There was a direct relationship between ∆G_0–30_ and gastric emptying (*r* = 0.47, *P* = 0.003). While there was no association of either ∆I_0–30_ (*r* = −0.16, *P* = 0.34) or fasting insulin (*r* = 0.21, *P* = 0.20), there were inverse relationships between the early insulin secretory response (*r* = −0.45, *P* = 0.004) and the DI (*r* = −0.33, *P* = 0.041), with gastric emptying. We conclude that gastric emptying is associated with both insulin secretion and the disposition index in subjects with normal glucose tolerance, such that when gastric emptying is relatively more rapid, both the early insulin secretory response and the disposition index are less. These findings should be interpreted as “hypothesis generating” and provide the rationale for longitudinal studies to examine the impact of baseline rate of gastric emptying on the prospective risk of type 2 diabetes.

## Introduction

There is a hyperbolic relationship between the insulin secretory response and insulin sensitivity during an oral glucose tolerance test, such that the composite of the two – the oral disposition index (DI) – is a constant (Utzschneider et al. 2009). Thus, normal glucose tolerance is maintained as long as the islet *β* cells have the capacity to upregulate insulin secretion and compensate for diminished insulin sensitivity. The failure of *β* cells to meet insulin secretory demand is fundamental to the pathogenesis of type 2 diabetes, so that with the progression from normal glucose tolerance to impaired glucose tolerance to type 2 diabetes there is a progressive shift in this curve downwards and to the left and a reduction in the disposition index (Utzschneider et al. 2009). A low disposition index at baseline was shown, in a community study of >600 people, to be predictive of progression to type 2 diabetes (Utzschneider et al. 2009) and, in type 2 diabetes, a lower disposition index is associated with the failure of a pharmacological intervention to slow progression of the disease process (Kahn et al. 2011).

Along with insulin secretion and sensitivity, gastric emptying is now recognized as a major determinant of oral glucose tolerance in health (Horowitz et al. 1993) and type 2 diabetes (Jones et al. 1996); when gastric emptying is more rapid, the initial glycemic response is greater (Marathe et al. 2013). The emptying of a liquid high in nutrients, such as 75 g glucose during an oral glucose tolerance test, approximates an overall linear pattern, but exhibits a wide interindividual variation of between 1 and 4 kcal/min in healthy subjects, a range which is even wider in type 2 diabetes because of the prevalence of both delayed and accelerated gastric emptying (Brener et al. 1983; Lin et al. 1989; Jones et al. 2001). Moreover, even relatively minor variations in gastric emptying can have a major effect on the postprandial glycemic excursion (Thompson et al. 1982; Horowitz et al. 1993; Jones et al. 1996). The exposure of the small intestine to nutrients, including glucose, as a result of gastric emptying triggers the release of the incretin hormones (glucose‐dependent insulinotropic polypeptide or GIP and glucagon‐like peptide 1 or GLP‐1) (Nauck 2011), which have a major impact on postprandial insulin secretion, accounting for 50–70% of insulin release in health during an oral glucose tolerance test (Nauck 2011). The secretion of both GIP and GLP‐1 is influenced by the rate of entry of glucose into the duodenum (Pilichiewicz et al. 2007; Marathe et al. 2014).

On the basis of the above evidence, we hypothesized that gastric emptying impacts on the insulin secretory response and the oral disposition index, and in this study, determined these relationships in a cohort with normal glucose tolerance.

## Materials and Methods

### Participants

Thirty‐nine healthy Caucasian volunteers (27 male and 12 female participants, mean age 30.1 ± 1.9 years, mean BMI 24 ± 0.6 kg/m^2^) were recruited through advertisement. Subjects with a history of gastrointestinal disease, other significant medical illness, or taking medication known to affect gastrointestinal motility, were excluded. The Royal Adelaide Hospital Human Research Ethics Committee approved the study protocol, which conformed to the principles of the Declaration of Helsinki, and all subjects provided written, informed consent before their participation.

### Protocol

Each subject fasted overnight (14 h for solids and 12 h for liquids) and attended the Department of Nuclear Medicine, PET and Bone Densitometry (Royal Adelaide Hospital) at 0800 h. While sitting in front of a gamma camera, each subject drank 350 mL water containing 75 g glucose labeled with 20 MBq ^99m^Tc‐sulfur colloid over 3 min. Time zero (*t* = 0) was taken as the end of drink consumption (Horowitz et al. 1993; Jones et al. 1996).

### Measurements of gastric emptying, blood glucose and plasma insulin

Gastric emptying data were acquired in 30 sec frames for the first 30 min, followed by 3 min frames for the subsequent 120 min. After ≈150 min, each subject drank 100 mL of water labeled with 5 MBq of ^99m^Tc‐sulfur colloid to enable a lateral image of the stomach to be recorded, which was used to derive correction factors for tissue attenuation (Collins et al. 1983). Data were also corrected for subject movement and radionuclide decay (Horowitz et al. 1993; Jones et al. 1996). From the gastric emptying curves the intragastric retention at *t* = 30, 60 and 120 min were determined, as well as the time for 50% emptying (*T*_50_) (Horowitz et al. 1993). The rate of gastric emptying, expressed as kcal/min, was derived from the *T*_50_.

Venous blood was obtained at 0, 30, 60, and 120 min. Plasma was separated by centrifugation and stored at −70°C for subsequent assays. Blood glucose concentrations were determined using a glucometer (Medisense Precision QID; Abbott Laboratories, Bedford, MA) and plasma insulin concentrations by ELISA (10‐1113 Mercodia, Uppsala, Sweden), with assay sensitivity of 1.0 mU/L and coefficient of variation 2.5% within assays and 7.4% between assays (Trahair et al. 2012). All subjects had normal glucose tolerance according to the World Health Organization criteria (blood glucose concentrations <6.1 mmol/L fasting, and <7.8 mmol/L at 2 h)(Alberti and Zimmet 1998).

### Calculation of early insulin secretory response, insulin sensitivity, and the oral disposition index

The insulin secretory response was estimated as the ratio of change in insulin to that of glucose at 30 min, represented as ∆I_0–30_/∆G_0–30_ (Retnakaran et al. 2009). Insulin sensitivity was estimated as 1/fasting insulin (Utzschneider et al. 2009). The disposition index was then calculated as ∆I_0–30_/∆G_0–30_ × 1/fasting insulin (Utzschneider et al. 2009).

### Statistical analysis

Blood glucose and plasma insulin concentrations at *t* = 0, 30, 60, and 120 min are shown as changes from baseline values (*t* = 0 min). Pearson's correlation was used to assess linear relationships between variables. Linear regression was used to assess the relationships of the change in glucose at 30 min, 1/fasting insulin, change in insulin at 30 min, early insulin secretory response (i.e., change in insulin relative to the change in glucose at 30 min) and the oral disposition index with gastric emptying. Our study was powered to examine the relationship between rate of gastric emptying and insulin secretory response. We calculated that a sample size of 36 would provide 80% power to detect a moderate correlation coefficient (*r* = 0.45) with an *α* = 0.05. Data are presented as mean values ± standard error margin (SEM), with statistical significance accepted at *P* < 0.05.

## Results

All subjects tolerated the study well and there were no adverse events. In all cases, gastric emptying approximated an overall linear pattern and the mean rate was 1.5 ± 0.06 kcal/min (mean ± SEM). Blood glucose levels (mmol/L) at baseline, 30, 60, and 120 min were 4.9 ± 0.1, 8.4 ± 0.2, 7.9 ± 0.3, and 5.7 ± 0.2, respectively (mean ± SEM).

### Relationships of changes in glucose and insulin at 30 min, and insulin sensitivity, with gastric emptying (Fig. 1A–C)

**Figure 1:**
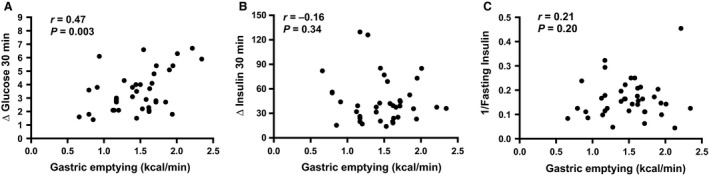
Relationships between (A) change in blood glucose from *t* = 0–30 min, (B) change in plasma insulin from *t* = 0–30 min and (C) 1/fasting insulin with gastric emptying expressed as kcal/min based on *T*_50_ in subjects with normal glucose tolerance. *T* = 0 min was the sample taken just prior to, and *t* = 30 min the sample taken 30 min after, consuming the glucose drink.

While the change in blood glucose at 30 min (∆G_0–30_) was related directly to gastric emptying (*r* = 0.47, *P* = 0.003), there was no significant relationship of the latter with the change in plasma insulin at 30 min (∆I_0–30_) (*r* = −0.16, *P* = 0.34). There was also no relationship between insulin sensitivity (1/fasting insulin) and gastric emptying (*r* = 0.21, *P* = 0.20).

### Relationships of early insulin secretory response and the oral disposition index with gastric emptying (Fig. 2A and B)

**Figure 2:**
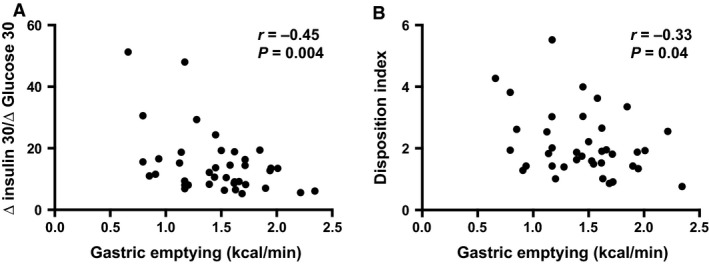
Relationships between (A) early insulin secretory response, and (B) oral disposition index with gastric emptying expressed as kcal/min based on *T*_50_ in subjects with normal glucose tolerance.

Both the early insulin secretory response (∆I_0–30_/∆G_0–30_) (*r* = −0.45, *P* = 0.004) and the oral disposition index (∆I_0–30_/∆G_0–30_ × 1/fasting insulin) (*r* = −0.33, *P* = 0.041) were related inversely to the rate of gastric emptying.

## Discussion

We have evaluated, in subjects with normal glucose tolerance, the relationships of glycemia, early insulin secretory response and oral disposition index during a 75 g oral glucose tolerance test with gastric emptying, quantifying the latter with the “gold standard” technique of scintigraphy. We confirmed that the early rise in blood glucose was related directly to the rate of gastric emptying. Novel, and important, observations are that both the early insulin secretory response and the oral disposition index, are inversely associated with gastric emptying.

We estimated the early insulin secretory response as the rise (change) in insulin relative to the change in glucose over 30 min following ingestion of oral glucose as in previous studies (Utzschneider et al. 2009). In health, the stimulation of insulin secretion by an oral/enteral glucose load primarily reflects the direct action of glucose on the *β* cell and the release of the incretin hormones, GIP and GLP‐1, which are both insulinotropic when blood glucose levels are elevated above ≈8 mmol/L, but not during euglycemia (Dupre et al. 1973; Schmidt et al. 1985). As noted previously (Horowitz et al. 1993), there was no relationship between the increase in plasma insulin at 30 min and gastric emptying, which in part, is likely to reflect the latency of the plasma insulin response to an increase in blood glucose. The magnitude of the elevation in blood glucose was above the threshold required for GIP and GLP‐1‐stimulated insulin secretion in ≈50% of the cohort, and changes in incretin secretion may, accordingly, also be relevant. Studies by ourselves and others (Pilichiewicz et al. 2007; Trahair et al. 2012; Marathe et al. 2014) have established that patterns of GIP and GLP‐1 release have different relationships with the rate of glucose entry to the duodenum. In health, when gastric emptying is <2 kcal/min (in our cohort, the mean gastric emptying was ≈1.5 kcal/min and no subject had an emptying rate ≥3 kcal/min), GIP, rather than GLP‐1, appears to be the dominant incretin; this cannot be the case in type 2 patients, because the insulinotropic effect of GIP, but not GLP‐1, is markedly attenuated (Nauck et al. 1993). Both GLP‐1 and insulin secretion are markedly increased when duodenal glucose delivery exceeds 3 kcal/min (Pilichiewicz et al. 2007; Trahair et al. 2012). We would hypothesize that in healthy subjects, the relationship between incretin‐mediated insulin secretion and gastric emptying after oral glucose will be nonlinear, so that when the rate of gastric emptying increases from ≈1–3 kcal/min, there is a relative reduction in the early insulin secretory response, but when gastric emptying is ≥3 kcal/min, the increase in insulin levels will be much greater (related to substantially greater GLP‐1 secretion), and potentially, adequate to compensate for the increase in blood glucose, so that the early insulin response (∆I_0–30_/∆G_0–30_) is constant. Further studies are indicated to address this hypothesis.

The observed inverse relationship between the oral disposition index and gastric emptying (Fig. 2B) is consistent with the relationship between the early insulin secretory response and gastric emptying, particularly given the absence of a relationship between insulin sensitivity and gastric emptying. Accordingly, in healthy when the early insulin secretory response is reduced, the oral disposition index is lower.

The limitations of our study should be recognized: the sample size was relatively small, and we measured glucose concentrations in blood rather than plasma (in general, the latter values are slightly higher). The insulin secretion was assessed indirectly. Moreover, the plasma insulin response is influenced by hepatic insulin extraction (Kautzky‐Willer et al. 1996; Rudovich et al. 2004), which we did not quantify; and in this regard measurement of C‐peptide would have been optimal. While we studied subjects with normal glucose tolerance, we recently reported that the relationships of gastric emptying with the early glycemic response (30 and 60 min) during an oral glucose tolerance test are stronger in subjects with impaired glucose tolerance and type 2 diabetes compared with normal glucose tolerance (Marathe et al. 2015). Given the marked impairment in *β*‐cell function in these states and the loss of an insulinotropic action of GIP in type 2 diabetes (Nauck et al. 1993), the relationships of the early insulin secretory response and disposition index with gastric emptying may well be stronger than we observed in health, but this needs to be confirmed. An implication of our observations is that relatively more rapid gastric emptying would predispose to type 2 diabetes, as has been suggested (Phillips et al. 1991; Marathe et al. 2015), not only because the initial glucose excursion is greater, but also because the early insulin secretory capacity is relatively diminished, so that compensation is inadequate. Interestingly, “early” type 2 diabetes has been associated with more rapid gastric emptying (Phillips et al. 1991, 1992), and the latter has also been observed in certain ethnic groups known to be predisposed to type 2 diabetes (Phillips 2006). If this is the case, slowing of gastric emptying, either by dietary (Gentilcore et al. 2006; Ma et al. 2009) or pharmacological strategies (e.g., “short‐acting” GLP‐1 agonists) (Linnebjerg et al. 2008; Lorenz et al. 2013), may potentially slow the progression of type 2 diabetes.

In conclusion, in individuals with normal glucose tolerance, both the early insulin secretory response and oral disposition index are inversely associated with the rate of gastric emptying. Our findings provide a rationale for longitudinal studies to determine the impact of gastric emptying on the risk of type 2 diabetes. Whether similar relationships are observed in individuals with diminished glucose tolerance and type 2 diabetes remains to be determined.

## Conflict of Interest

None declared.
